# 9G4+ Antibodies Isolated from HIV-Infected Patients Neutralize HIV-1 and Have Distinct Autoreactivity Profiles

**DOI:** 10.1371/journal.pone.0085098

**Published:** 2013-12-26

**Authors:** Danielle C. Alcéna, James J. Kobie, Denise A. Kaminski, Alexander F. Rosenberg, Jonelle L. Mattiacio, Matthew Brewer, Stephen Dewhurst, Carrie Dykes, Xia Jin, Michael C. Keefer, Ignacio Sanz

**Affiliations:** 1 Department of Microbiology and Immunology, University of Rochester Medical Center, Rochester, New York, United States of America; 2 Division of Allergy, Immunology and Rheumatology, University of Rochester Medical Center, Rochester, New York, United States of America; 3 Division of Infectious Diseases, University of Rochester Medical Center, Rochester, New York, United States of America; 4 Lowance Center for Human Immunology and Division of Rheumatology, Department of Medicine, Emory University, Atlanta, Georgia, United States of America; Emory University School of Medicine, United States of America

## Abstract

Potent HIV-1 specific broadly neutralizing antibodies (BNA) are uncommon in HIV infected individuals, and have proven hard to elicit by vaccination. Several, isolated monoclonal BNA are polyreactive and also recognize self-antigens, suggesting a breach of immune tolerance in persons living with HIV (PLWH). Persons with systemic lupus erythematosus (SLE) often have elevated levels of autoreactive antibodies encoded by the VH4-34 heavy chain immunoglobulin gene whose protein product can be detected by the 9G4 rat monoclonal antibody. We have recently found that levels of these “9G4+” antibodies are also elevated in PLWH. However, the putative autoreactive nature of these antibodies and the relationship of such reactivities with HIV neutralization have not been investigated. We therefore examined the autoreactivity and HIV neutralization potential of 9G4+ antibodies from PLWH. Results show that 9G4+ antibodies from PLWH bound to recombinant HIV-1 envelope (Env) and neutralized viral infectivity *in vitro*, whereas 9G4+ antibodies from persons with SLE did not bind to Env and failed to neutralize viral infectivity. In addition, while 9G4+ antibodies from PLWH retained the canonical anti-i reactivity that mediates B cell binding, they did not display other autoreactivities common to SLE 9G4+ antibodies, such as binding to cardiolipin and DNA and had much lower reactivity with apoptotic cells. Taken together, these data indicate that the autoreactivity of 9G4+ antibodies from PLWH is distinct from that of SLE patients, and therefore, their expansion is not due to a general breakdown of B cell tolerance but is instead determined in a more disease-specific manner by self-antigens that become immunogenic in the context of, and possibly due to HIV infection. Further studies of 9G4+ B cells may shed light on the regulation of B cell tolerance and interface between the generation of specific autoreactivities and the induction of antiviral immunity in persons living with HIV.

## Introduction

Broadly neutralizing antibodies (BNA) develop in a minority of HIV-infected persons, and typically only after several years of infection; moreover, they are mostly absent following administration of candidate HIV vaccines [1-3]. The underlying reasons for this are likely complex, but have been proposed to include the fact that several BNA are polyreactive and also recognize self-antigens [4,5]. Thus, it has been hypothesized that breakdown of B cell tolerance may be important, if not required, for the generation of broadly neutralizing antibody responses in at least some individuals [5,6]. 

However, while previous studies may have been limited in scope, persons living with HIV (PLWH) with strong BNA responses have not been reported to a have higher incidence of autoimmune diseases. One possible explanation is that the putative breach in B cell tolerance may be incomplete. To examine this question, we have studied the properties of a well-characterized population of autoreactive antibodies; 9G4+ antibodies, found both in persons with SLE and in PLWH [7]. These antibodies represent a subset of autoreactive antibodies encoded by the VH4-34 heavy-chain gene, which are recognized by an anti-idiotypic reagent, the 9G4 rat anti-human monoclonal antibody, and have previously been found to be at higher frequencies in persons with SLE [8-11].

Isolated 9G4+ antibodies from persons with SLE bind apoptotic cells [12], a common SLE autoreactivity [13-15], but they also bind to naïve B cells through recognition of N-acetyllactosamine moieties of B220/CD45R [16,17]. In addition, serum levels of 9G4+ antibodies are correlated with overall disease severity in persons with SLE [8]. We therefore conducted studies to compare the functional and autoreactive properties of 9G4+ antibodies from PLWH and from persons with SLE. As part of these experiments, we also examined the anti-HIV properties of these antibodies as a follow-up to our recent study showing that circulating 9G4+ antibody levels are correlated with HIV broadly neutralizing serum reactivity in PLWH [7]. Our current data show that 9G4^+^ antibodies from PLWH possess HIV envelope (Env)-binding and virus-neutralizing activity, but have restricted autoreactivity, and lack many of the key self-reactive properties of 9G4+ antibodies from persons with SLE. This is a remarkable finding given the strong structural constraints imposed on 9G4 antibodies by their universal expression of the intrinsically autoreactive VH4-34 gene segment, and the high degree of censorship of this population of antibodies from the memory B cell and serum IgG compartment in healthy individuals [18,19]. Hence, our results bear significant implications for the selection of potentially protective autoreactive antibodies in the context of HIV infection. 

## Materials and Methods

### Ethics Statement

All human samples were obtained using procedures and methods in accordance with the Declaration of Helsinki and approved by the University of Rochester Medical Center (URMC) Research Subjects Review Board. All subjects provided written informed consent. 

### Clinical Samples

HIV-infected patients in this study were obtained from the URMC Infectious Diseases Clinic between 2004 and 2011. Samples from patients diagnosed with systemic lupus erythematosus (SLE) were obtained from the URMC Allergy, Immunology, and Rheumatology Clinic. SLE patients met the American College of Rheumatology revised criteria for the classification of disease. The SLE patients were selected if they fulfilled at least 4 of the 11 criteria of the American College of Rheumatology for the classification of SLE; had at least one serum autoantibody (either anti- dsDNA, Sm, RNP, Ro or La antibodies); and a Systemic Lupus Erythematosus Disease Activity Index (SLEDAI) score greater than 4 or a clinical flare, defined by the SELENA-SLEDAI index. Samples from normal donors were obtained at the URMC. Peripheral blood mononuclear cells, plasma, and sera were isolated and cryopreserved as previously described [20]. 

### IgG Purification

IgG purification was done using Aspire Protein G Tips (Thermo-Scientific, Rockford, IL) according to manufacturer’s guidelines. Briefly, each sample was diluted in 1x sample buffer and added to the color-coded tubes in series filled with wash binding buffer, wash buffer and elution buffer, respectively. Each subjects' elution fractions were pooled separately, before buffer exchange into PBS. Fractions were then screened and quantified by ELISA. All steps were performed at room temperature (RT). 

### 9G4 Purification

IgG Ab fractions were fractionated by 9G4 using modifications to the Protein G, IgG Plus Orientation kit (Thermo-Scientific, Rockford, IL). Briefly, columns were placed in 15 ml tubes and centrifuged at 100 RCF for 1 min to drain storage buffer and then washed twice (re-suspended in 5 ml binding/wash buffer then centrifuged 1 min at 100 RCF). Next columns were capped and allowed to bind with 6 mg/column of the rat anti-human 9G4 mAb, kindly provided by F.K. Stevenson (University of Southampton, Southampton, United Kingdom). Column coupling was done by placing columns on a rotator (mimicking hand-overhand rotations) for 3 h at RT. Columns were then cross-linked using manufacturer guidelines. After coupling, 9G4 coupled- protein G slurries were divided into additional columns (one per patient plasma sample). Each patient plasma sample was diluted in binding buffer (PBS, 0.1M phosphate, 0.15 M NaCl, pH 7.2) and added to the 9G4-coupled column and allowed to bind for 3 h at RT with rotation. Columns were then washed in binding buffer with centrifugation. The flow-through was collected (9G4- fractions). This was repeated until unbound antibodies were removed from the column and then elution buffer (Pierce IgG Elution Buffer, 21004) was added and column was again mixed with rotation for 5 min followed by centrifugation. The flow-through was collected (9G4+ fractions). All unbound fractions were pooled and elution fractions were pooled separately, before buffer exchange into PBS. Fractions were then screened and quantified by ELISA and used for subsequent studies.

### Total IgG, 9G4, and Viral ELISAs

For total IgG ELISA, plates (Nunc- Polysorb) were coated with 50 µl/well volume of goat anti human IgG Abs (Jackson Research, 109006097) at 2 µg/ml in PBS (Invitrogen), then incubated for 1 h at RT followed by 3 washes with wash buffer (PBS+0.1% Tween20). Blocking was then done for 20-30 minutes with RT Pierce Super Blocking Solution (Pierce, Cat. 37515), 200 µl/well at RT followed by 3 washes in wash buffer. Serially diluted assay standard, (Jackson ImmunoResearch, West Grove, PA. 009000003) and dilute samples (human sera or purified human serum antibodies) in sample diluent solution (3 parts PBS, 1 part Pierce Super Block Solution, plus 0.05% Tween 20) were added to plates at 50 µL/well before a 90 min incubation at RT. Plates were next washed and alkaline-phosphatase (AP) conjugated anti-IgG (Sigma-Aldrich, St. Louis, MO, A9544) diluted 1:30,000 was added at 50 µl/well for 1 h at RT. Plates were then washed again, Blue Phospho substrate (KPL, Gaithersburg, MD 508800) was added at 100 µl/ml following manufacturer guidelines, and optical density was measured at 650 nm. For 9G4 ELISA, plates were coated, washed and blocked as above. A serially diluted standard was included as a control (75D9, recombinant VH4-34 encoded monoclonal antibody). 9G4 antibodies were detected using biotinylated rat anti-human 9G4 mAb (diluted 1:3,000 from a 1mg/ml stock); this was added at 50 µl/well for 1 h at RT. Plates were then washed and incubated with 50 µl/ml of streptavidin-AP (Jackson Research I. 016050084) for 1 h at RT. Washing and detection of AP was performed as described above. For HIV gp140 ELISA, plates were coated with 2 µg/ml purified HIV-1 YU2 gp140 oligomeric envelope protein [21].. For Influenza virus- and CMV, ELISA plates were coated with 5 µg/ml of Influenza Virus Vaccine, Fluzone® (2008-2009, 2009-2010 and 2010-2011 formulations) or 1 µg/mL of HCMV AD 169 strain purified viral lysate (Advanced Biotechnologies, Inc., Columbia MD) diluted in PBS. 

### HIV-1 Neutralizing Activity

Clade B Tier 1 (SF162) pseudo-typed virus was incubated with heat-inactivated plasma and then added to TZM-bl indicator cells as previously described [22]. Neutralizing antibody titers were determined as the reciprocal plasma dilution at which luciferase expression was reduced relative to the assay internal controls.

### Apoptotic Lymphocyte Binding Assay

Apoptotic lymphocyte binding of antibodies was determined as previously described [12,23]. Briefly, Jurkat cells (J45.01) at 1x10^6^/mL were treated with 20 µM of the apoptosis-inducing drug, camptothecin (Sigma-Aldrich, St. Louis, MO) then incubated at 37 °C for 18 h, after which cells were washed twice (re-suspended in RPMI 1640 and centrifuged at 350 RCF for 7 min). Cells were then re-suspended in blocking solution (RPMI 1640 with 10% normal mouse serum and 10% normal rat serum), incubated for 10 min at RT and washed once. Samples were transferred from 5 ml tubes to FACS plates (BD Pharmingen, San Diego, CA) and washed once. Media was aspirated from the cell pellets before plasma or 9G4 fractionated antibodies were added for 30 min on ice. Next the cells were washed and stained with an antibody cocktail: Pacific Blue-CD3 (Pacific Blue-conjugated mouse anti-human CD3, SP34-2, BD 558124), APC-Cy7-CD19 (APC-Cy7-conjugated mouse anti-human CD19, SJ25C1, BD 557791), APC-CD27 (APC-conjugated mouse anti-human CD27, ebioscience, San Diego, CA, 17-0279-73), PE-IgD (PE-conjugated mouse anti-human IgD, IA6-2, BD 555779), and FITC-9G4 (FITC-rat anti-human Ig idiotype, Batch#3, 2008; F.K. Stevenson, U. of Southampton, UK) in the dark for 30 min on ice. Cells were then centrifuged at 350 RCF for 10 min at 4 °C, stained with LIVE/DEAD Fixable Aqua Dead Cell Stain Kit (Invitrogen, Grand Island, NY L34957) in the dark for 30 min on ice, washed and re-suspended in 0.05% paraformaldehyde. Simply Cellular Compensation Standard (Bangs Laboratories, Fishers, IN 550), ArC Amine Reactive Compensation Bead Kit (Invitrogen, A10346), and Rainbow Calibration Peak 6 particles (Spherotech, Lake Forest, IL RCP-30-5A-6) were used for single stain compensation controls, LIVE/DEAD compensation controls and application specific-settings, respectively. Live/Dead stain positive cells (dead/dying cells) were gated upon. For acquisition, ~1 million total events per sample were collected on an LSRII instrument (BD Biosciences) and analysis performed in a blinded manner using FlowJo software (Treestar, Inc., Ashland, OR). 9G4+ antibody binding to live Jurkat cells is not observed [12,23]. 

### B Cell Binding Assay

B cell binding was determined as previously described [12,16,23]. Briefly, normal tonsillar cells were thawed, washed, and re-suspended in blocking solution (RPMI 1640 with 10% normal mouse serum and 10% normal rat serum) and incubated for 10 min at RT followed by one wash. Samples were transferred from 5 ml tubes to FACS plates followed by one wash. Cells were aspirated before plasma or 9G4 fractionated antibodies were added for 30 min on ice. Next the cells were washed before they were stained with our B cell antibody cocktail and analyzed as described above (note that, in this case, we gated on the viable cell population). B cell binding by normal polyclonal purified human serum IgG is not observed [12,23]. 

### Cardiolipin and ANA ELISAs

Anti-cardiolipin (IgG, IgM, and IgA) ELISA (ALPCO Immunoassays, Salem, NH) and Anti- Nuclear Antigen (ANA) IgG ELISA (INOVA Diagnostics, Diego, CA) were performed according to manufacturer’s guidelines. Briefly, samples were diluted and then added to pre-coated plates. Plates were washed four times between each step of the protocol. For cardiolipin, sample readings of <11 U/ml were in the range of normal, 11-13 U/ml were considered a borderline positive, while >13 U/ml were considered a true positive. For ANA, sample readings of <20 Units were negative, 20-60 Units positive and >60 Units strong were considered strong positives.

### Anti-Nuclear Antigen (ANA) Indirect Immunofluorescence (IFA)

ANA IFA (Inova Diagnostics, Diego, CA) was performed according to manufacturer’s guidelines. Briefly, samples were diluted and then added to pre-coated slides. Purified antibodies were added at 100 µg/ml. Visualization of IFA signal was done using a fluorescence microscope (495 nm excitation filter and 515 nm emission filter)

### Auto-Antigen Microarray

Auto-antigen microarray analysis was conducted by the Proteomics Core at UT Southwestern University, Dallas, TX as previously described [24]. Briefly, serum/antibody samples were incubated with the auto-antigen array and antibody binding was detected at 532 nm (Cy3 labeled anti-IgG) and 635 nm (Cy5 labeled anti-IgM or IgA). Tiff image files were generated, and analyzed using Genepix Pro 6.0 software. Net fluorescence intensities were defined as the spot minus background fluorescence intensity; data obtained from duplicate spots were averaged. Signal-to-noise ratio (SNR) was used as a quantitative measure of the ability to resolve true signal from background noise; SNR equal or greater than 3 was considered to indicative of a true signal. 

### Statistical Analysis

Statistical significance was assessed using unpaired Mann-Whitney test (unless otherwise noted in text). Statistical analyses were performed using Prism software version 6.0b (Graph Pad Software, La Jolla, CA) and significance was taken as p≤ 0.05 (unless otherwise noted in text).

## Results

### Apoptotic Cell Binding by 9G4+ Antibodies from PLWH

**Figure 1:**
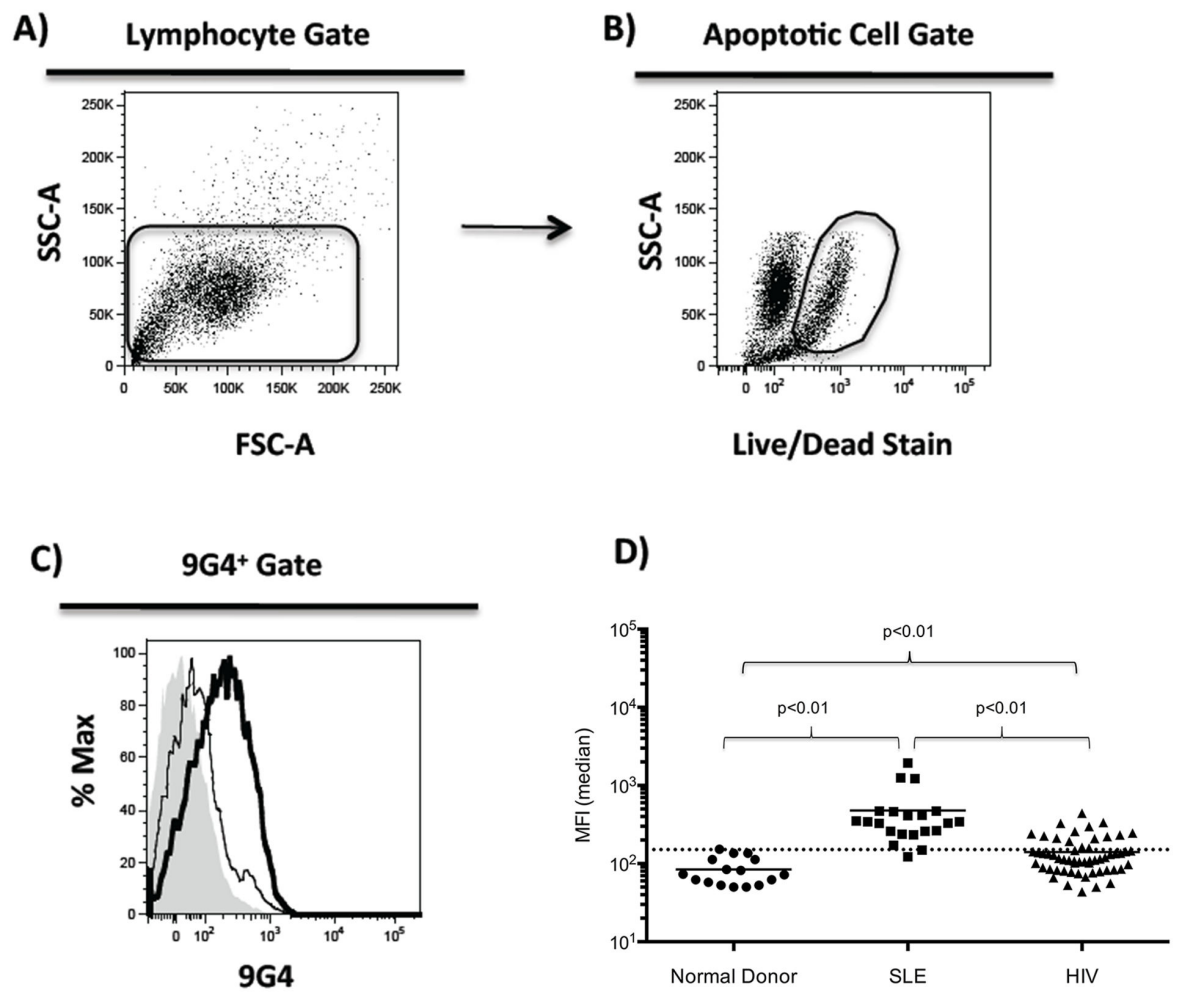
9G4+ antibodies from HIV-1 infected patients bind apoptotic lymphocytes. Unfractionated serum from normal donors (n=16), SLE (n=21) and HIV- infected (n=57) were used in apoptotic binding assays. (**A**-**C**) The Jurkat Human T cell line was treated with camptothecin to induce apoptosis then incubated with patient serum. Serum binding to cells was detected using FITC- labeled 9G4 rat anti-human monoclonal antibody. Apoptotic Cell Gate was drawn around cells stained positive for viability dye, representing the dying cell population. (**C**) The shaded histogram represents cells incubated with PBS in the absence of patient serum. The thin-line represents the antibody binding from a normal serum donor and the Bold-line is representative of 9G4+ antibody binding from an HIV infected patient. (**D**) Data from all samples are plotted. The dashed-line represents the normal donor mean plus 2 standard deviations. Significance determined by Mann-Whitney test.

Serum antibodies to apoptotic lymphocytes are a common feature of SLE autoreactivity [12-14]. We therefore tested the binding of 9G4+ serum antibodies from our cohort of 57 PLWH (Table **1**) for the ability to bind to apoptotic lymphocytes. We found that sera from 13 out of 57 (23%) PLWH compared to nearly all SLE patients (19 out of 21) exhibited binding to apoptotic cells (Figure **1**). Moreover, the level of reactivity was significantly lower in HIV patients compared to SLE. Subgroup analysis did not indicate an association of other factors (viral load, CD4 count, time since diagnosis, ART status, total 9G4+ serum antibody titer) with apoptotic binding (data not shown). These results suggest that apoptotic cell binding mediated by 9G4+ antibodies is a feature of a relatively small subset of PLWH and that overall, this 9G4 autoreactivity is significantly attenuated in PLWH. 

**Table 1:** Demographics and clinical characteristics of HIV-infected patient samples.

**Characteristic**	**HIV-1 infected (n=57)**
Mean age (years, range)	44 (21-75)
*Gender*	
Male	35
Female	22
*Race*	
White	35
Black	22
Unknown	4
*Ethnicity*	
Hispanic	6
Not Hispanic	51
Mean Time since HIV Dx (years, range)	9 (0.01-23.7)
CD4 Count (cells/uL±SD)	638 (±279)
Viral Load (copies/mL±SD)	11,084 (±33,559)
*ARTStatus*	
On	14
Off	13
Never	30
Serum 9G4+ antibody (mean relative units±SD)	176 (±369)

### 9G4+ Antibodies Purified from PWLH Bind to HIV Envelope and Neutralize HIV

**Figure 2:**
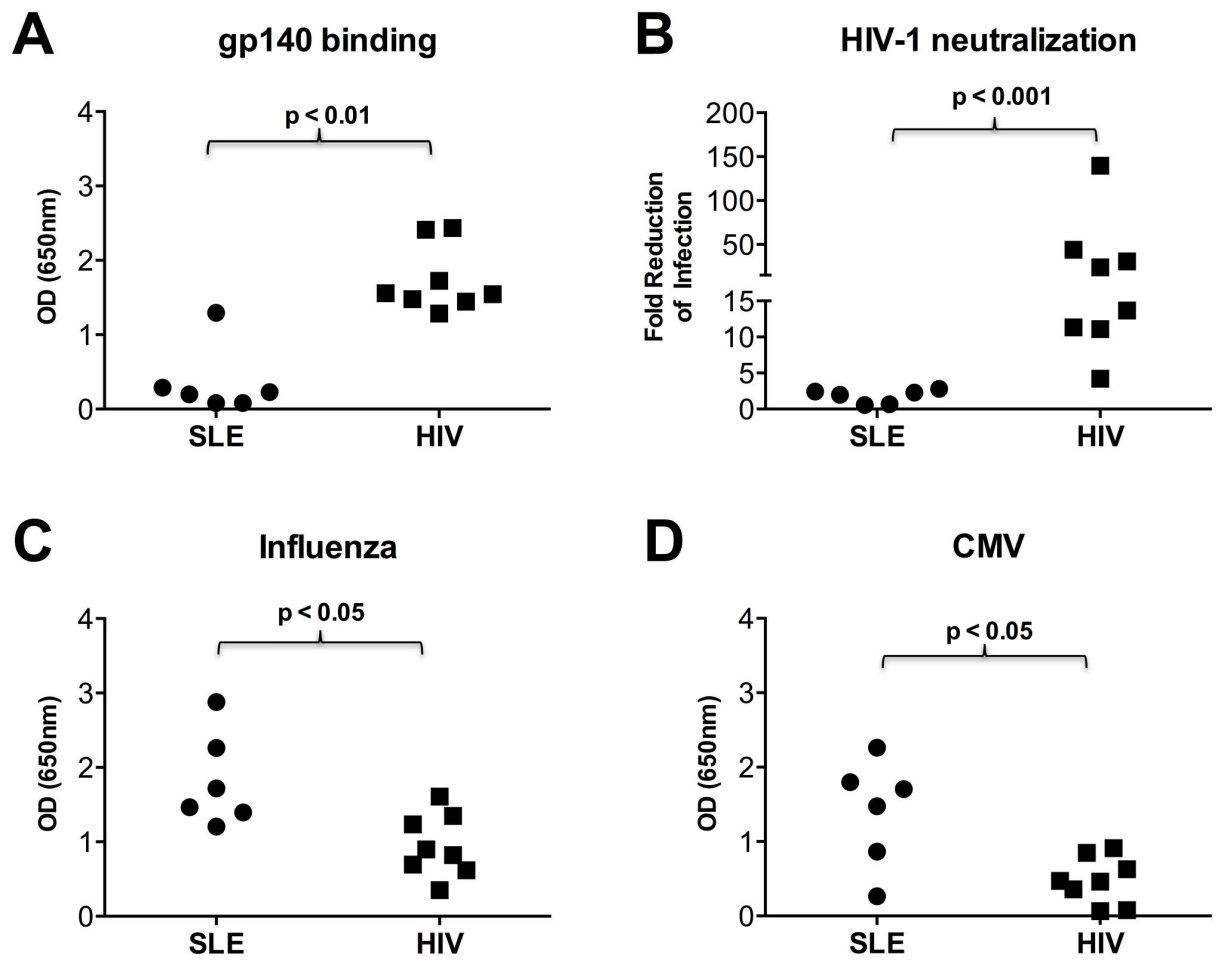
9G4+ antibodies purified from HIV- infected patients bind to HIV gp140 and neutralize HIV-1. 9G4+ antibodies isolated from SLE patients (n=6) and PLWH (n=8) normalized to equal concentrations, were serially diluted in triplicate and added to ELISA plates coated with oligomeric YU2 gp140 (**A**) Influenza vaccine (**C**) or CMV lysate (**D**) and binding detected by anti-IgG. (**B**) 9G4+ antibodies were tested for inhibition of pseudotyped HIV-1 SF-162 infection of TZM-bl cells. Infectivity was measured by luciferase assay, and relative inhibitions were calculated after normalizing to internal controls. Each symbol represents the mean value of a patient. Significance determined by Mann-Whitney test.

We previously demonstrated a positive correlation between serum 9G4+ antibody levels and HIV BNA activity in PLWH [7]. Therefore, we purified 9G4+ antibodies from the plasma of a subset of PLWH with elevated 9G4+ antibodies (Table **2**). These purified 9G4+ antibodies were then evaluated for their ability bind to HIV Env. Purified 9G4+ antibodies from PLWH reacted strongly with HIV-1 gp140, whereas 9G4+ antibodies from most SLE patients did not (p<0.01, Figure **2A**). Interestingly, one SLE patient sample was able to recognize HIV-1 Env (Figure **2A**). 

**Table 2:** Characteristics of the patient subset for 9G4 studies.

**Characteristic**	**HIV-1 infected (n=8)**
Mean age (years, range)	46 (23-71)
*Gender*	
Male	5
Female	3
*Race*	
White	4
Black	3
Unknown	1
*Ethnicity*	
Hispanic	1
Not Hispanic	7
Mean Time since HIV Dx (years, range)	16 (4.2-22.8)
CD4 Count (cells/uL±SD)	504 (±279)
Viral Load (copies/mL±SD)	1,504 (±3,035)
*ARTStatus*	
On	3
Off	2
Never	3
Serum 9G4+ antibody (mean relative units±SD)	826 (±475)

We next assed the ability of 9G4+ antibodies from PLWH to neutralize infectivity of HIV-1 SF-162 [25]. We found that 9G4+ antibodies isolated from PLWH (n=8) neutralized *in vitro* infection, whereas 9G4+ antibodies from persons with SLE did not (n=6) (p<0.01, Figure **2B**). 

Common viral infections like CMV can stimulate the production of VH4-34-encoded (9G4+) IgM [26,27]. We therefore tested whether 9G4+ antibodies from PLWH might have activity against multiple viruses, including CMV and influenza. We found that 9G4+ IgG from PLWH had similar (low) levels of reactivity to influenza virus antigens (Figure **2C**) and CMV lysate (Figure **2D**), when compared to 9G4+ IgG from persons with SLE. 

### 9G4+ Antibody Fractions Isolated from PLWH Have B Cell Binding Autoreactivity

**Figure 3:**
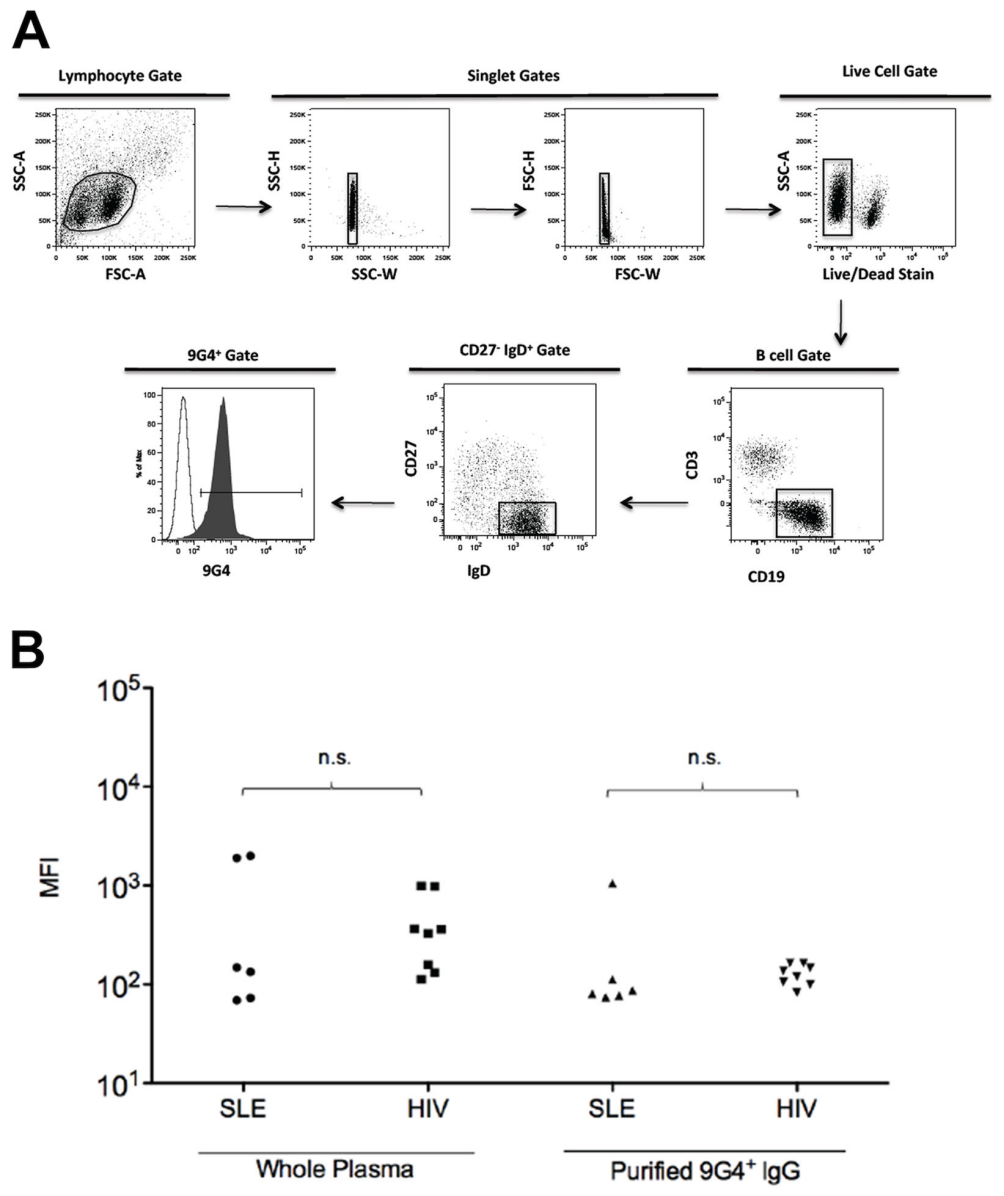
9G4+ antibodies from SLE and HIV-infected patients bind B cells. Tonsil cells were incubated with either patient plasma, purified 9G4+ antibodies from patients’ plasma or a positive control 9G4+ monoclonal antibody then stained with fluorescently -labeled antibody cocktails. (**A**) Gating strategy: lymphocytes were gated based on size and granularity followed by an exclusion of doublets. Dead cells were excluded using a viability stain then live cells were then gated by expression of CD3 and CD19. CD3-CD19+ B cells were then further divided by IgD and CD27 markers. The open histogram represents the no antibody source control (PBS alone). The solid histogram is representative of 9G4+ antibody binding B cells. (**B**) B cell binding of 9G4+ antibodies purified from SLE (n=6) and HIV- infected (n=8) patients was assessed. Significance was determined by Mann-Whitney test.

Most 9G4+ antibodies display intrinsic autoreactivity due to their expression of VH4-34 heavy chains. This canonical autoreactivity is characterized by binding to glycoproteins expressing N-Acetyl-lactosamine glycans including the I/i blood group antigens and a CD45/B220 glycoform expressed on the surface of naïve B cells [16]. We therefore used flow cytometry to measure binding by our purified 9G4+ IgG to tonsillar B cells from healthy human donors (Figure **3**). This analysis revealed that 9G4+ IgG from PLWH exhibited similar B cell binding activity as 9G4+ IgG from SLE patients (Figure **3**). 

### 9G4+ IgG from PLWH Has Reduced Cardiolipin Reactivity and Lacks Antinuclear Antibody (ANA) Activity

**Figure 4:**
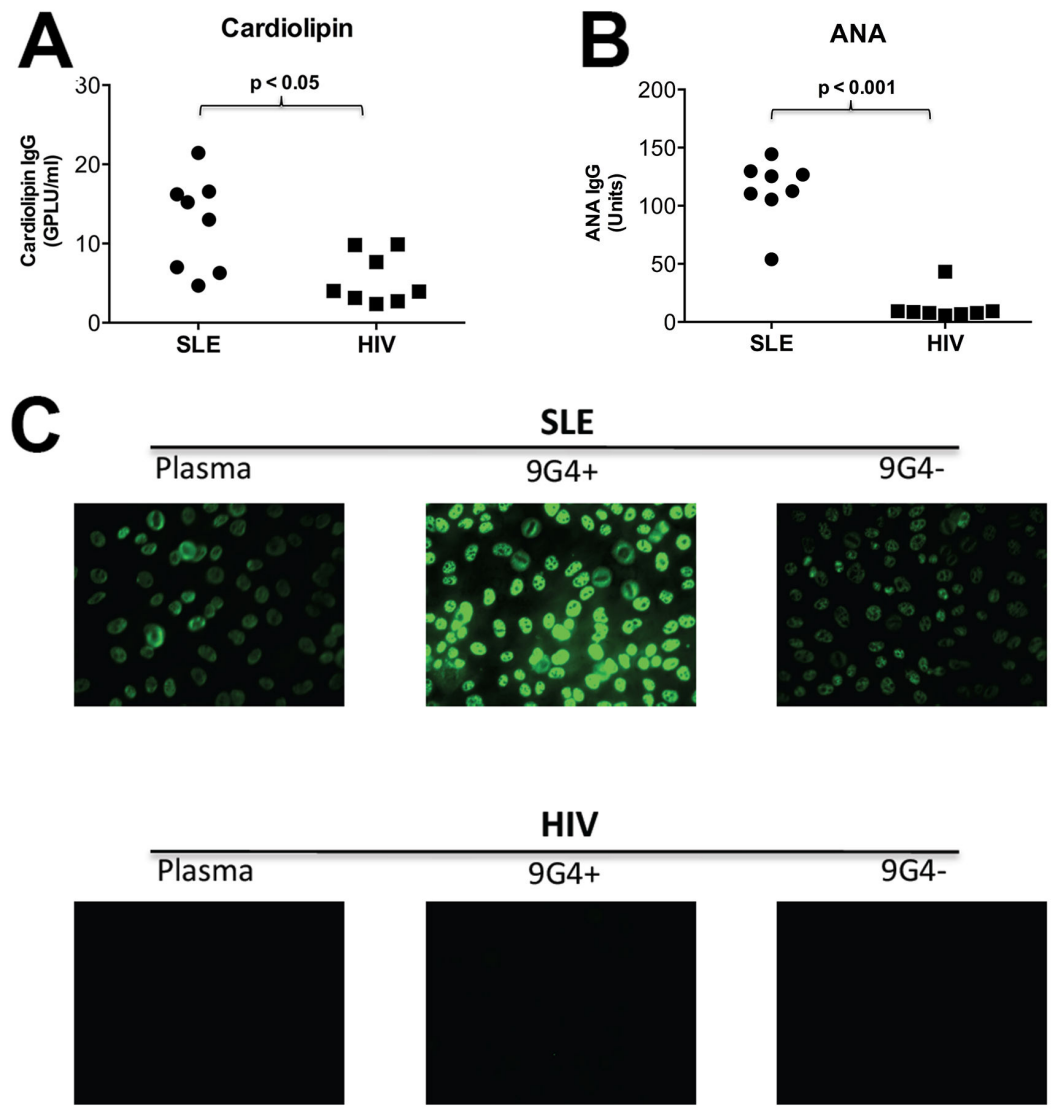
9G4+ antibodies isolated from HIV-1 infected patients exhibit less Cardiolipin and ANA autoreactivity than 9G4+ isolated from SLE patients. Purified 9G4+ antibodies (from SLE [n=8] and HIV-1 infected patients [n=8]) were added to Cardiolipin (**A**) and HEp2 lysate (**B**) coated ELISA plates. Anti-IgG was used to detect antibody binding. (**B**) Cardiolipin IgG (GPLU/ml) and (**C**) ANA IgG (arbitrary units) were calculated based on manufacturer guidelines, and data were plotted. Each dot represents a sample. Significant differences between HIV and SLE patients were observed in 9G4+ antibody binding to cardiolipin and ANA as determined by Mann-Whitney test. (**C**) Slides coated with HEp-2 cells were incubated with patient antibodies, detected with FITC conjugated anti-IgG, and visualized with a fluorescence microscope. Dilutions of patient plasma were selected based on manufacturer guidelines while fractionated samples were used at 100 µg/ml. Representative images for unfractionated plasma, 9G4+ and 9G4- antibody fractions from an SLE patient and HIV patient are presented.

We next asked if the 9G4+ antibodies from PLWH could bind to specific host antigens, which is also a feature of 9G4+ antibodies in SLE. Our results show less cardiolipin binding of 9G4+ antibodies from PLWH compared with 9G4+ antibodies from persons with SLE. Only two out of 8 PLWH (25%) had moderate levels (>11 GPLU/ml) of cardiolipin-specific antibodies (Figure **4A**). Antinuclear antibody (ANA) binding by ELISA was completely absent in the 9G4+ IgG from PLWH compared to that of SLE controls (Figure **4B**). This lack of ANA activity by 9G4+ IgG from PLWH was confirmed *via* immunofluorescence assay (IFA) detected using HEp2 target cells (Figure **4C**). Collectively, these findings show that 9G4+ IgG from PLWH has less cardiolipin reactivity and ANA activity, as compared to 9G4+ antibodies from persons with SLE, further suggesting distinct characteristics of 9G4+ IgG from PLWH compared to those isolated from SLE patients. 

### Depleting 9G4^+^ Antibody from Plasma of PLWH Reduces Autoreactivity

**Figure 5:**
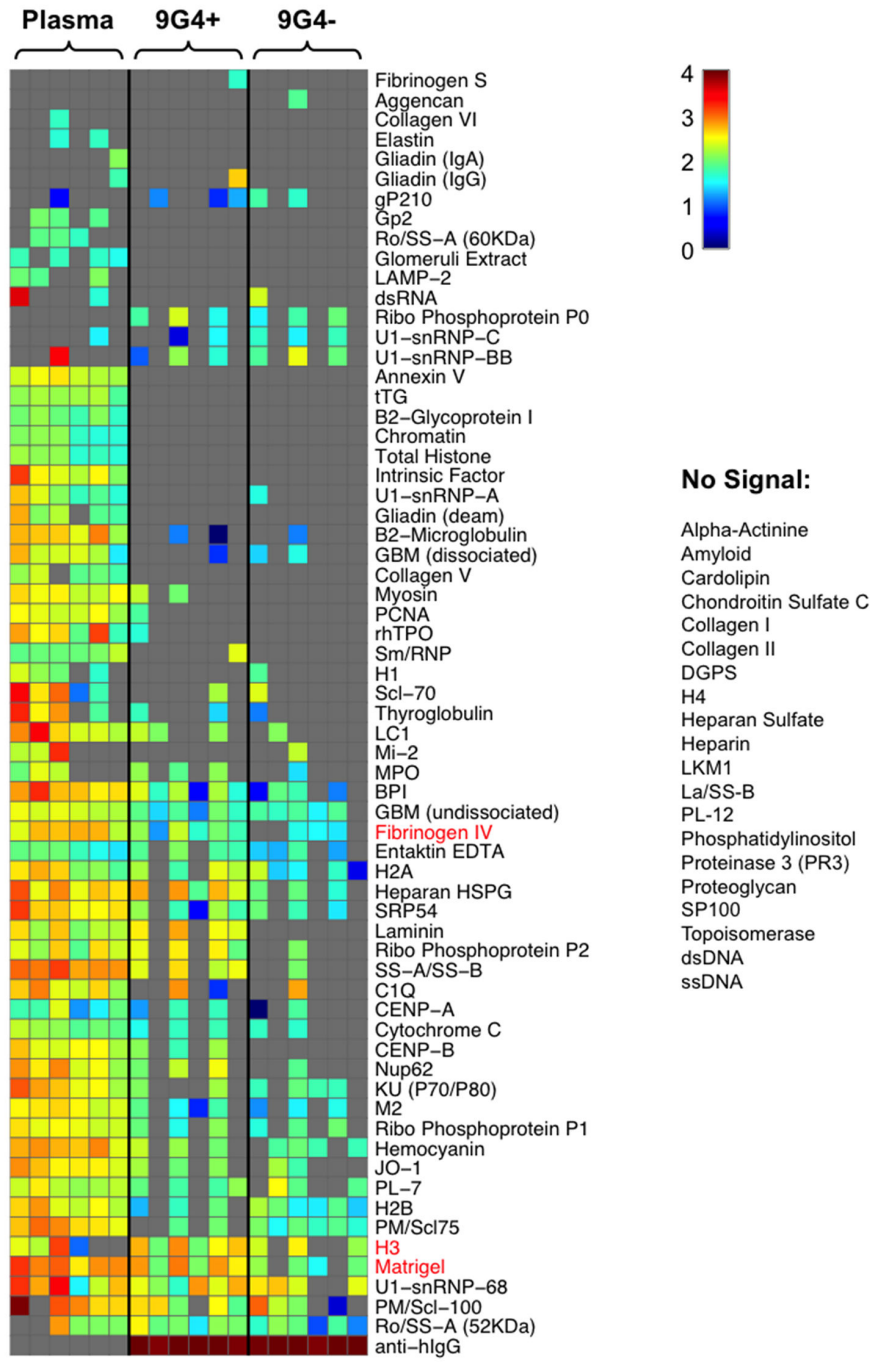
Auto-antigen microarray profiles of 9G4+ IgG isolated from HIV-infected patients. Plasma, 9G4+ and 9G4- antibodies were screened for IgG binding to 85 auto-antigens. Columns represent HIV samples, rows represent antigens and color maps to log_10_ signal (gray indicates lower limit of detection, LLOD). Columns are organized by sample type (unfractionated plasma, 9G4+, 9G4-) and subject order within groups is the same for each type. Rows are clustered based on Euclidean distance to facilitate visualization. Antigens that resulted in no signal are listed to the right of the heat map. Antigens in red indicate higher 9G4+ binding relative to 9G4- based on a Wilcoxon signed-rank test at p<0.05. Average signal data where either the signal intensity was greater than 100 or the signal-to-noise ratio was greater than 2 were considered to be above the LLOD; these data were background-subtracted (based on PBS control per antigen) and then log_10_ transformed.

Because the 9G4+ IgG autoreactivity profiles differed between PLWH and SLE patients, we next examined the overall profile of antibody autoreactivity of PLWH using an autoantigen microarray, containing approximately 100 glomerular-derived antigens that have been previously shown to differentiate clinical SLE subpopulations [28,29]. Plasma from PLWH (n=6) had detectable reactivity to 62 out of the 85 (72.9%) autoantigens used, but not to cardiolipin, dsDNA or La/SS-B (Figure **5**). When plasma was separated into 9G4+ and 9G4- fractions, a higher overall rate of autoreactivity was detected within the 9G4+ fraction. This included significantly increased reactivity to selected extracellular matrix proteins (Fibrinogen IV, H3, and Matrigel), as well as an elevated, but not statistically significant reactivity to Ro/SS-A and SS-A/SS-B (Figure **5**). Interestingly, considerable autoreactivity remained in the 9G4- fraction, suggesting non- 9G4+ sources of autoreactivity. 

## Discussion

9G4+ antibodies are highly autoreactive in SLE, a disease in which patient serum titers correlate with disease activity [8,16,17]. Moreover, in SLE, 9G4 antibodies contribute the majority of autoantibodies reacting against B cells and apoptotic cells [12,16,17] and the latter type of autoreactivity, which is found in approximately 60% of all SLE patients and in >80% of SLE patients with elevated titers of serum 9G4 antibodies, correlates with the presence of lupus nephritis [12]. These observations have broadened the spectrum of autoreactivity of 9G4 autoantibodies in SLE and thus, they provide the foundation to better understand the autoreactivity of 9G4+ antibodies recently described in PLWH [7], and the overall regulation of B cell tolerance in persons infected with HIV-1. To begin to address these questions, we examined the autoreactivity of 9G4+ antibodies from PLWH, and compared them to similar antibodies from persons with SLE. It is important to note that recent structural studies performed in our laboratory with a large panel of mAbs derived from single SLE B cells have established that the canonical autoreactivity of 9G4 antibodies, anti-B cell binding (BCB), is fully dependent on the conservation of a hydrophobic patch (HP) encoded by the germline sequence of the VH4-34 framework-1 region, this HP is not required for anti-apoptotic cell binding (APCB), and that in fact, this type of VH4-34-encoded autoreactivity as well as anti-dsDNA binding frequently increases when the HP is disrupted by *in vitro* mutagenesis, a manipulation that also abolishes the expression of the 9G4 idiotype [23]. Therefore, it is likely that the analysis of 9G4+ antibodies may significantly underestimate the contribution of HP-independent VH4-34-encoded autoreactivity to both autoimmune disease and HIV-triggered autoantibody responses. 

Our studies revealed that the autoreactivity of 9G4+ IgG from PLWH was significantly different than that of autoimmune SLE patients. Thus, PLWH have much lower levels of 9G4+ APCB since only a fraction of the sera from our cohort of PLWH (13/57 subjects, or 23%) contained 9G4+ IgG reactive with apoptotic lymphocytes and did so with a rather low degree of autoreactivity. This result was not due to lower levels of global serum 9G4 antibodies and is consistent with our observations in SLE patients [12]. More importantly, it reflects the fact that that APCB, which is independent of the expression of the germline-encoded FR1 HP, is not an inherent feature of all 9G4+ antibodies but may instead, depend on the expression of charged CDR3-H regions in a fraction of 9G4+ antibodies [23]. A detailed follow-up analysis of the 8 subjects with the highest levels of serum 9G4+ antibodies demonstrated that purified 9G4+ antibodies from this group of PLWH exhibited essentially identical B cell binding activity to 9G4+ antibodies from persons with SLE. Yet, similar to APCB, the 9G4+ antibodies from PLWH exhibited a very pronounced difference from 9G4+ antibodies from SLE patients in cardiolipin binding and ANA activity, a reactivity that we have also found to be HP-independent in our SLE mAb studies [23]. 9G4+ IgG from persons with SLE had strong reactivity with cardiolipin and robust ANA activity; in contrast, 9G4+ IgG from PLWH had weak ANA activity and little reactivity to cardiolipin. This preferential expansion of 9G4+ antibodies with HP-dependent autoreactivity strongly suggests a profound difference in the degree to which B cell tolerance is breached in persons with SLE, versus persons infected with HIV. Whether these differences are due to differences in genetic susceptibility to generalized tolerance breakdown and/or to differential availability of selecting autoantigens in both conditions cannot be differentiated by our current data. Thus, it is possible that defective apoptotic cell clearance, an important defect with a genetic basis in SLE might not be pronounced in most HIV patients thereby precluding the exposure of immunogenic intracellular and nuclear antigens to the immune system. Alternatively, the differences observed in the type of 9G4+ autoreactivity expanded in PLWH could be also explained on the basis of preferential selection of HP-dependent autoreactivity. This model is supported by the differences we found between the two patient categories in the ability of their serum 9G4+ antibodies to bind HIV-1 Env as 9G4+ antibodies from most SLE patients failed to bind to HIV-1 Env, whereas 9G4+ antibodies from our cohort of PLWH showed robust binding to HIV-1 Env. Importantly, since the fractionated 9G4+ antibodies from PLWH were also able to neutralize infectivity of a Tier-1 clade B HIV-1 strain (SF162), these results warrant further testing of 9G4+ antibodies against more neutralization resistant Tier-2/3 HIV-1 strains and non-clade B strains in future studies to directly determine the potency and breadth of the 9G4+ antibodies. Collectively, these data suggest that 9G4+ antibodies in PLWH may be expanded as part of an HIV-specific B cell response and may contribute to antibody-mediated recognition and neutralization of HIV-1. This possibility is of particular interest given the nature of the antigens involved in this type of autoreactivity and their implications for HIV infection and vaccination. Indeed, we initially demonstrated using enzymatic manipulation and immunoprecipitation that the canonical HP-dependent autoreactivity of lupus IgG 9G4 autoantibodies is directed against N-acetyl-lactosamine (NAL) glycans expressed by the I/i blood group antigens and other multiple mammalian glycoproteins including B220, thereby accounting for the BCB activity of these autoantibodies [16]. More recently, we have provided direct evidence for this autoreactivity using 9G4 mAbs from SLE patients and extensive high-density glycan arrays [23]. It is therefore plausible that this germline-encoded anti-glycan canonical autoreactivity may be responsible for the expansion of anti-HIV Env 9G4+ autoantibodies and if so, this property would render the stimulation of 9G4+ cells an attractive target for HIV vaccines. Further study to determine if 9G4+ antibodies recognition of HIV-1 envelope is mediated through binding to glycan-dependent epitopes should be conducted. Along these lines, understanding the molecular basis of self and viral glycan reactivity, and potential cross-reactivity between the two, study of 9G4 autoantibodies should prove a highly informative approach to developing new HIV immunogens capable of at least priming HIV reactive B cells found at high frequency in the pre-immune B cell repertoire of the general population as 9G4+ B cells account for 5-10% of both naïve B cells and unswitched IgM+ natural and marginal zone B cells [18,19], a population that in mice has been shown to contribute the major fraction of anti-gp41 MPER antibodies [30]. Our findings suggest that immunization strategies capable to drive these naïve and IgM+ memory B cells into germinal center reactions might successfully induce protective anti-HIV antibodies without undesirable autoimmunity. Given the intrinsic anti-glycan autoreactivity of germline VH4-34 antibodies, vaccination approaches designed to trigger 9G4+ B cells might be able to induce protective antibodies that do not require high levels of somatic hypermutation, an elusive goal for conventional vaccination schemes. However, it remains to be determined whether the very autoreactivity of 9G4+ antibodies may pose a risk of clinical autoimmunity if these antibodies are elicited by vaccination in healthy subjects. This possibility is illustrated by the enrichment in poly-autoreactivity shown for the 9G4+ serum fraction in our antigen microarray studies. On the other hand, substantial experimental evidence indicates that the generation of autoantibodies is not sufficient for clinical autoimmunity and therefore, it is possible that in subjects lacking the multiple genetic and environmental hits that are presumed to be responsible for the development of clinical autoimmunity, this intervention might prove safe and successful.

Full elucidation of these critical questions will require additional studies, such as in-depth molecular and functional comparison of the VH4-34 immunoglobulin repertoire and binding properties of SLE and PLWH, which is outside of the scope of the present study. 

Overall, our findings provide support for the notion that normal B cell tolerance is impaired in at least some persons infected with HIV-1, and suggest that autoreactive B cells may contribute to host protection, through the secretion of 9G4+ antibodies that recognize and neutralize HIV-1. These findings may have important implications for HIV-1 vaccine design and for our understanding of both B cell tolerance and the pathogenesis of HIV-1 infection. 
